# Evaluating the impact of simulation on patient outcomes in pediatric and neonatal critical care: a systematic review and critical appraisal of the evidence

**DOI:** 10.1007/s00431-026-06925-3

**Published:** 2026-04-26

**Authors:** Anna Zanin, Elisa Poletto, Santiago Mencia, Orsola Gawronski, Gilles Jourdain

**Affiliations:** 1 Department of Women, Children, and Adolescents, Interdisciplinary Coordination and Care Center for Rare and Complex Diseases in Children and Adolescents, Geneva, Switzerland; 2 SIMDream, Department of Women’s and Children’s Health, University of Padua, Padua, Italy; 3 Pediatric Intensive Care Unit, San Bortolo Hospital, Vicenza, Italy; 4 Pediatric Intensive Care Unit, Hospital Gregorio Maranon, Madrid, Spain; 5 Professional Development, Continuing Education and Research Unit, Bambino Gesu Children’s Hospital IRCCS, Rome, Italy; 6 Division of Pediatric and Neonatal Critical Care and Transportation, AP-HP Paris Saclay University-Antoine Beclère Hospital, Clamart, France; 7 Simulation Centre LabForSIMS, Paris Saclay University, Le Kremlin-Bicêtre, France

**Keywords:** Simulation, Pediatric Kirkpatrick model, Resuscitation, Pediatric intensive care unit

## Abstract

While the literature widely supports a positive relationship between simulation-based education (SBE) and learning outcomes, its impact on patient outcomes remains less clearly understood. Our aim was to comprehensively survey the landscape of simulation-based research (SBR) that evaluates patient outcomes (Kirkpatrick Level 4) in the context of pediatric and neonatal critical care. A systematic search of MEDLINE, Cochrane Library, CINAHL, and ERIC was conducted through December 2024. We included RCTs, non-RCTs, and before–after studies focusing on Kirkpatrick Level 4 outcomes. Quality was appraised using the RoB 2 and ROBINS-I tools. A total of 2481 articles were screened. Sixteen studies met the inclusion criteria. Most were prospective (63%) and single-center (69%), with only three RCTs (19%). All included studies (100%) reported Level 4 outcomes; however, a critical distinction emerged between system-level and patient-level results. While 75% (12/16) of studies reported positive impacts on Level 4a (system processes), such as protocol adherence or response times, only 50% (8/16) demonstrated significant improvements in Level 4b (direct patient outcomes), such as mortality or complication rates. In addition, all included studies were judged to have a high or critical risk of bias.

*Conclusions*: Although SBE effectively optimizes clinical processes (Level 4a), a significant evidence gap remains regarding its direct impact on patient health (Level 4b). Current SBR often lacks the methodological rigor—specifically the use of randomized designs—needed to isolate SBE as the primary driver of clinical improvement. Future research must prioritize high-quality, multicenter trials targeting patient-oriented metrics to bridge this translational gap.

**Table:** 

**What is Known:** • *SBE has been extensively developed in pediatric and neonatal critical care settings; however, its impact on healthcare professional training and on patient outcomes remains less well understood.* **What is New:** • *There is a scarcity of rigorous Level 4 evidence linking SBE to patient outcomes in pediatric and neonatal critical care.* • *We provide a methodological roadmap advocating for mixed-methods and multicenter designs to bridge this clinical evidence gap.*

**Supplementary information:**

The online version contains supplementary material available at https://doi.org/10.1007/s00431-026-06925-3.

## Introduction

Advancements in surgical and medical care within pediatric and neonatal critical care have introduced increasingly complex procedures; yet, training methodologies remain largely unstandardized, often unstructured, and heavily reliant on the resources available at individual institutions [1–6]. This presents a significant challenge, particularly given the declining frequency of invasive procedures, which limits trainees’ clinical exposure and may contribute to adverse patient outcomes [7, 8]. The introduction of simulation-based education (SBE) aims to address these challenges by providing an effective approach to enhance provider performance [6–8]. However, while some studies show SBE improves technical and nontechnical skills, its impact on patient outcomes remains less definitive [9–11].

To accurately evaluate this relationship, we must distinguish between the educational intervention itself (SBE) and the research framework used to measure its efficacy (simulation-based research (SBR)) [12].

While SBE and SBR should ideally work in tandem to demonstrate how simulation-driven training translates into improved patient outcomes, their relationship remains complex. This is often hindered by gaps between evolving medical treatments and the lack of standardized training protocols [13, 14].

**Fig. 1:**
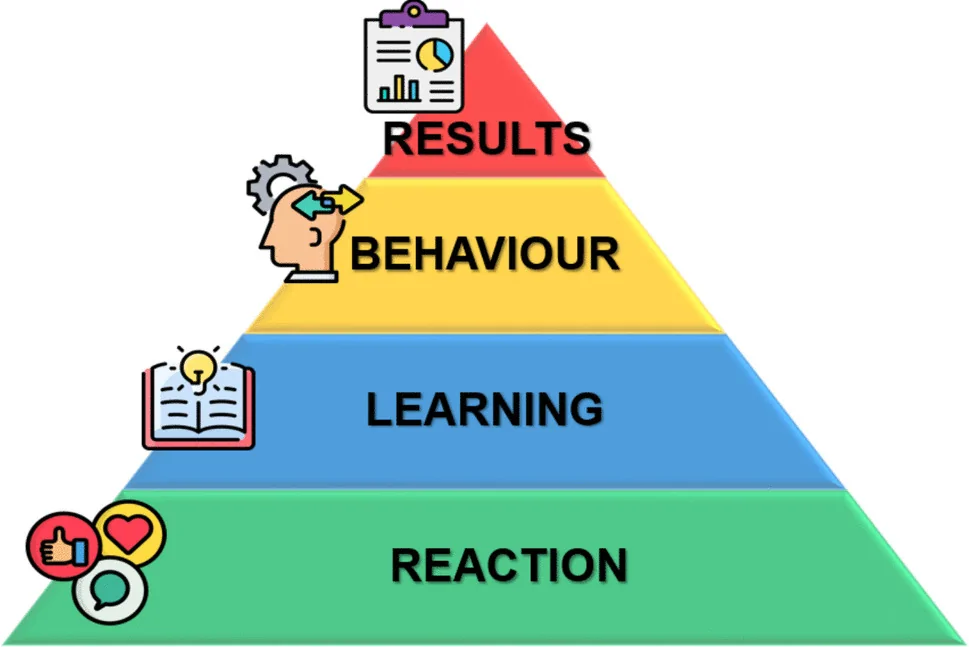
Overview of Kirkpatrick’s four-level model of training evaluation

The Kirkpatrick model (Fig. 1) is a widely used four-level framework for evaluating the effectiveness of educational interventions, including SBE [15]. It comprises four levels: Level 1 (reaction), which assesses participants’ immediate responses and satisfaction with the training; Level 2 (learning), which measures the knowledge, skills, and attitudes acquired by the participants; Level 3 (behavior), which evaluates the application of learned behaviors in clinical practice; and Level 4 (results) examines the broader impact of the training on the organization, specifically patient care and outcomes in this case.

In the context of healthcare and medical education, Kirkpatrick’s Level 4 outcomes focus on the ultimate effect of the organizational or systemic performance [16, 17]. To provide a deeper analysis of simulation’s efficacy, some authors divide this level into Level 4a (organizational/system-level) and Level 4b (patient-level) [15]. Level 4a assesses improvements in healthcare delivery and operational efficiency—such as optimized emergency response times, increased protocol adherence, or reduced medical errors. Conversely, Level 4b focuses on direct clinical consequences, including patient morbidity, mortality, and cost-effectiveness [15]. This distinction is fundamental to our review; it allows us to differentiate between improvements in the clinical process (system-level) and measurable therapeutic benefits (patient-level).

This study aims to systematically review the existing literature to evaluate the impact of SBE on clinical outcomes in pediatric and neonatal critical care patients. Specifically, it focuses on (P) pediatric and neonatal critical care, (I) SBE interventions, (C) traditional training/no training, and (O) patients’ outcomes. The primary outcome of interest corresponds to Kirkpatrick’s Level 4.

To date, there is no comprehensive review that systematically maps and critically appraises the current state of high-level SBR within pediatric and neonatal critical care patients and clinical settings [18–20].

## Methods

### Study design

The research question was structured using the Population Intervention Comparison Outcomes (PICO) format (see table S1, supplementary material 1). This systematic review was conducted in accordance with Cochrane methodology [21, 22] and reported following the Preferred Reporting Items for Systematic Reviews and Meta-analyses (PRISMA) statement guidelines [23]. The protocol was uploaded on the PROSPERO website in August 2024 (ID: CRD42024583975). Institutional Review Board approval was not required for this study.

### Study eligibility criteria

Studies were included if they met the following criteria: (1) the study design was a RCT, no-RCT, or before–after study; (2) participants were healthcare providers or trainees performing clinical care tasks and system processes in the pediatric intensive care units (PICUs) or in the neonatal intensive care units (NICUs); and (3) the study evaluated one or more clinical outcomes potentially improved by the intervention, corresponding to Level 4 of the Kirkpatrick model.

### Outcome measures

Eligible outcome categories included any documented clinical improvement or reduction in patient complications, with all outcomes required to be based on care delivered to actual patients. The primary outcome of this systematic review was to systematically evaluate and synthesize the existing evidence on the effectiveness of SBE interventions in improving patient-related clinical outcomes, corresponding to Kirkpatrick Level 4, in pediatric and neonatal critical care settings. The secondary outcome was to identify and categorize the specific types of Level 4 clinical outcomes reported—such as mortality rates, procedure-related complications, and patient safety events—in studies successfully assessing SBE in this population.

### Search methods

A comprehensive literature search was conducted in MEDLINE (1946–2024), the Cochrane Library (Cochrane reviews, 2014–2024), CINAHL (1984–2024), and ERIC (1966–2024), using strategies developed in consultation with a professional librarian. Search terms included “simulation,” “simulation-based research,” “simulation-based education,” “patient outcomes,” “pediatric critical care,” and “neonatal critical care” (for search strings per database see table S2, supplementary material 1). No limits or language restrictions were applied. All searches were last updated on December 31, 2024. Additionally, the reference lists of included studies were manually screened to identify any further relevant publications.

### Study selection, data collection, and quality assessment

**Fig. 2:**
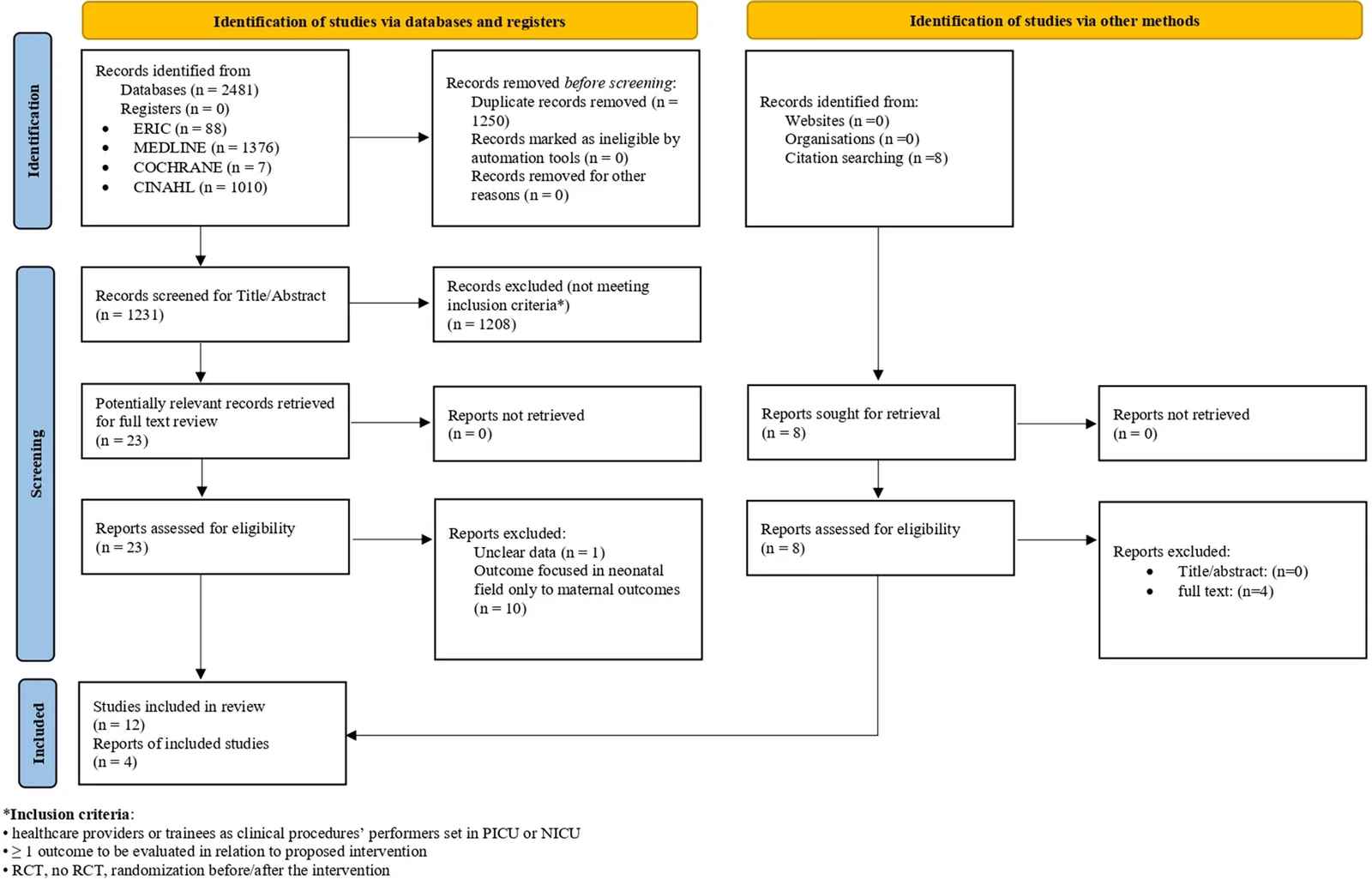
Prisma flow diagram of study selection: this figure outlines the stepwise process of study identification, screening, eligibility assessment, and final inclusion in accordance with PRISMA2020 guidelines

Two reviewers (A.Z. and E.P.) initially screened studies identified through electronic database searches, excluding studies based on design (e.g., editorials, letters, case reports, case series, and narrative reviews). Then, three investigators (A.Z., E.P., and G.J.) independently reviewed the titles and abstracts of the remaining records; excluding those that did not meet the inclusion criteria. Potentially relevant non-English articles were translated to determine study eligibility. The full texts of the remaining 23 articles were manually reviewed by four investigators (A.Z., E.P., O.G. and S.M.), who were not blinded to the journal titles, institutions, or authorship (Fig. 2). Data extraction was performed using a standardized, pilot-tested extraction form. We specifically extracted data regarding (1) study characteristics (year, country, and setting); (2) population details (neonatal vs. pediatric); (3) the specific SBE intervention; and (4) the nature of the Level 4 outcomes reported. To ensure consistency, outcomes were explicitly coded as 4a or 4b based on whether the primary metric was a process-based system measure or a direct patient health indicator. Any discrepancies in categorization were resolved through a consensus meeting with the senior investigator (G.J.).

RCTs were evaluated using the Revised Cochrane Risk-of-Bias (RoB) 2 tool [21], while no-RCT were evaluated using the Risk of Bias in Non-randomized Studies of Interventions-Version2 (ROBINS-I-V2) tool [22]. Disagreements regarding the quality assessment of studies were resolved through consensus with the senior investigator (G.J.), as per the data collection procedure. Interrater reliability was assessed with Cohen’s kappa coefficient, a statistical measure of interrater agreement for qualitative items. Based on the 95% confident interval, values less than 0.5 indicate poor agreement, between 0.5 and 0.75 moderate agreement, between 0.75 and 0.9 good agreement, and greater than 0.90 excellent agreement.

### Analysis, data synthesis, and heterogeneity

Due to the significant heterogeneity in study designs, variable selection, and analytical methods across the included articles, a quantitative meta-analysis was not feasible. Instead, we conducted a narrative synthesis to integrate the findings. We systematically mapped the extracted data onto the Kirkpatrick framework by dividing results into two primary thematic categories: Level 4a (system-level outcomes), such as adherence to resuscitation protocols and team response times, and Level 4b (patient-level outcomes), such as mortality rates and procedural complication frequencies. This qualitative approach allowed us to synthesize evidence from diverse clinical contexts while accounting for the methodological variations identified during our quality assessment.

## Results

### Study selection and characteristics

After the removal of duplicate studies, 1231 potentially relevant publications were identified for screening. After screening titles and abstracts, we identified 23 studies for full-text review, of which 16 of them met the inclusion criteria and were included in the final analysis (Fig. 2). Tables 1 and 2 report all the studies following the reoperationalization of the outcome levels based on the Kirkpatrick model, with particular emphasis on Level 4, which was further subdivided into Level 4a (system-level process metrics) and Level 4b (actual patient outcomes). Table 1 provides detailed information on the study’s characteristics, participants, interventions, outcomes, results, and key findings. Table 2 offers a structured mapping of each study’s outcomes to L1, L2, L3, 4a, and 4b, along with reported significance levels and effect sizes.

**Table 1:** Overall characteristics of the studies included

Study(author, year)	Setting and designduration	Population/sample	Intervention	Key outcomes and results
Studies focused on improvements in system-level process (Level 4a)				
Kessler et al. (2011) [9]	USA (New York); single-center prospective, RCT12 months	56 pediatric residents	Simulation-based deliberate practice for infant lumbar puncture (LP) skills	Improved odds of obtaining CSF in the next clinical LP; reduction in traumatic LPs (25% vs. 43%)
Su et al. (2014) [10]	USA (Washington); Single-centerRetrospective, before–after40 months	43 pediatric cardiac patients (ECPR)	High-fidelity simulation to define roles and create an “ideal rapid deployment” checklist	Significant reduction in ECMO deployment time; improved team coordination and role clarity
Theilen et al. (2017) [31]	UK (Edinburgh); single-centerProspective, before–after48 months	79 staff;139 unplanned PICU admissions	Weekly in-situ simulation for a Pediatric Medical Emergency Team (pMET)	Sustained improvement in response to critical illness; significant reduction in unplanned PICU admissions and bed days
Studies focused on improvements in patient-level outcome (Level 4b)				
Andreatta et al. (2011) [28]	USA (Michigan); single-centerProspective, mixed-methods48 months	252 mock code responses	Randomly called mock codes with immediate debriefing over 48 months	CPA survival rates increased to ~ 50% within 1 year; sustained impact on patient survival
Scholtz et al. (2013) [24]	USA (Philadelphia); single-centerProspective; before–after24 months	524 inpatient nurses	Mastery learning CVC “dress rehearsal” education program	Improved nurse psychomotor skills; temporally associated decrease in hospital-wide CLABSI rates
Mduma et al. (2015) [36]	Tanzania; single-centerProspective; before–after24 months	9708 newborns (pre/post-cohorts)	Frequent brief (3–5 min weekly) on-site HBB simulation training	Significant decrease in 24-h neonatal mortality (from 11.1 to 7.2 per 1000)
Gibbs et al. (2017) [26]	USA (New York); single-centerProspective; before–after4 months	99 providers;18 MRSA patients	30-min in situ simulation to resolve latent safety threats during an MRSA outbreak	Sustained improvement in hand hygiene; no new MRSA colonizations for 6 months post-intervention
Schmidt et al. (2018) [34]	Laos; multicenterRetrospective; before–after32 months	2216 neonatal patients	Simulation-based neonatal teaching program twice yearly	Decrease in mortality/morbidity from infection and asphyxia; however, overall mortality rate increased due to prematurity
Kumar et al. (2018) [32]	Australia; multicenterProspective; mixed-methods60 months	508 staff; ~ 27,000 births	Implementation of PROMPT simulation package for obstetric emergencies	Improved recognition of postpartum hemorrhage; lower shoulder dystocia rates; increased staff confidence
Lenguerrand et al. (2019) [30]	UK (Scotland); multicenter prospective; stepped-wedge RCT30 months	87,204 births	Unit-level implementation of PROMPT training for all maternity staff	Moderate but nonsignificant reduction in Apgar < 7 at 5 min; suggested cost-effectiveness in reducing litigation
Sawyer et al. (2019) [29]	USA (Seattle); single-centerRetrospective; quasi-exp32 months	332 professionals; 29 simulations	Monthly 2-h high-fidelity ECPR simulations in ICU settings	Improved adherence to activation protocols and faster activation; survival increased but not statistically significant
Balachander et al. (2020) [33]	India (Bengaluru); single-centerRetrospective; before–after13 months	Nurses and PG doctors in NICU	Simulation training for IV cannulation and aseptic non-touch technique	Improved success rates and aseptic awareness; reduction in BSI, PLABSI, and CLABSI rates
Herrick et al. (2021) [11]	USA (Philadelphia); single-centerRetrospective; before–after38 months	34 neonates; 1182 intubations	Individualized airway bundle (AB) designed and tested via simulation	Significant reduction in severe tracheal intubation-associated events (TIAEs); increased use of video laryngoscopy
Chitkara et al. (2023) [25]	USA (California); multicenter prospective; before–after34 months	2,626 VLBW Infants	12-month in situ simulation program for neonatal resuscitation	No significant difference in survival without CLD or secondary outcomes
Studies focused on improvements in system-level process (Level 4a) and in patient-level outcome (Level 4b)				
Walker et al. (2015) [35]	Mexico; multicenterProspective; cluster RCT31 months	450 providers; 50,589 births	PRONTO: simulation-based low-cost obstetric/neonatal emergency training	40% reduction in neonatal mortality at 8 months; decrease in C-section incidence
McKeta et al. (2021) [27]	USA (Charleston); single-centerRetrospective; before–after42 months	108 patients	Specialized pediatric cardiac response team and C-CHEWS score training	Reduction in cardiac arrests, mortality, and unplanned transfers to the cardiac ICU

*AHA-PALS* American Heart Academy-Pediatric Advanced Life Support, *AB* airway bundle, *AV* audiovisual, *BSI* blood stream infections, *BRE* blinded rater evaluation, *CC* chest compression, *C-CHEWS* cardiac children’s hospital early warning score, *CLABSI* central line associated blood stream infection, *CLD* chronic lung disease, *CPA* cardiopulmonary arrest, *CPR* cardiopulmonary resuscitation, *CVC* central venous catheter, *ECMO* extracorporeal membrane oxygenation, *ECPR* extracorporeal cardiopulmonary resuscitation, *ECLS* extracorporeal life support, *HBB* helping babies breathe, *GW* gestational week, *IHCA* intrahospital cardiac arrest, *ICU* intensive care unit, *IV* intravenous, *LP* lumbar puncture, *MRSA* methicillin-resistant *Staphylococcus aureus,* *NA* not available, *NICU* neonatal intensive care unit, *NMR* neonatal mortality rate, *NR* not reported, *OSCEs* observed structured clinical examinations, *PG* postgraduate doctor in pediatrics, *PICU* pediatric intensive care unit, *PLABSI* peripheral line–associated blood stream infection, *pMET* pediatric medical emergency team, *PROMPT* practical obstetric multiprofessional training, *RCT* randomized control trial, *SPE* standardized parent evaluation, *TIAEs* adverse tracheal intubation-associated events, *VL* video-laryngoscope, *VLBW* very low birth weight

**Table 2:** Panoramic of the studies according to Kirkpatrick levels

Study	Outcome measures	Mapped level	Significance/effect size
Studies focused on improvements in system-level process(Level 4a)			
Kessler et al. (2011) [9]	• Self-reported clinical success, change in confidence, and knowledge • Infant LP skills (measured via OSCE on simulator) • Rates of traumatic clinical LPs	• L1 (reaction)/L2 (learning) • L2 (learning) • L4a (system-level process)	• Self-reported success was improved Confidence and knowledge remained high • Same improvement in OSCE performance at the beginning, 6, and 8 months • Not statistically significant (25% vs. 43%)
Su et al. (2014) [10]	• Improvement of multidisciplinary quality of the team • Reduction of median deployment time of ECMO	• L3 (behavior/process) • L4a (system-level process)	• Subjectively observed improved coordination and smoother deployment of ECMO in real-life • Significantly faster deployment of ECMO
Theilen et al. (2017) [31]	• Improvements in hospital response to deteriorating patients and reduced time to first staff response • Reduction of unplanned PICU admissions and PICU bed days	• L3 (behavior/process) • L4a (system-level process)	• Improvement in hospital response to evolving critical illness and reduced time from patient deterioration to first staff response • Significant reduction in the number of unplanned PICU admissions Reduction of related PICU bed days year on year
Studies focused on improvements in patient-level outcome(Level 4b)			
Andreatta et al. (2011) [28]	• Residents’ confidence in performing resuscitations • Patient CPA survival rates	• L1 (reaction) • L4b (patient-level outcome)	• Improvement in learner-perceived value and confidence • Substantial, sustained impact on CPA survival rates
Scholtz et al. (2013) [24]	• Provider knowledge, self-confidence, and psychomotor skills on mannequins • Provider clinical performance on real patients • Hospital-wide CLABSI rate	• L2 (learning) • L3 (behavior/process) • L4b (patient-level outcome)	• Improvement of nurse’s knowledge, self-confidence, and psychomotor skill performance on mannequins • Procedural competence on real patients in clinical practice improved • Temporally associated decrement of hospital CLABSI rates
Mduma et al. (2015) [36]	• Reduction in 24-h neonatal mortality	• L4b (patient-level outcome)	• Significant decrease in neonatal mortality at 24-h (from 11.1/1000 to 7.2/1000; *p* = 0.040)
Gibbs et al. (2017) [26]	• Enhanced estimate of the culture of safety • Hand hygiene compliance • New MRSA colonization/infection	• L1 (reaction) • L3 (behavior/process) • L4b (patient-level outcome)	• Enhanced estimate of the culture of safety • Improvements in hand hygiene, sustained for a 6-month period • No new MRSA colonization/infection
Schmidt et al. (2018) [34]	• Reduction of neonatal mortality and morbidity due to infection and asphyxia • Total neonatal mortality rate at 24-h	• L4b (patient-level outcome) • L4b (patient-level outcome)	• Decrease of neonatal mortality and morbidity as well as case fatality rates • Significant decrease (from 11.1/1000 to 7.2/1000; *p* = 0.040)
Kumar et al. (2018) [32]	• Change in management of postpartum hemorrhage • Clinical outcomes (rate of shoulder dystocia) and neonatal deaths	• L3 (behavior/process) • L4b (patient-level outcome)	• Change in the management of postpartum hemorrhage by early recognition and intervention • The rate of shoulder dystocia was significantly lower (*p* = 0.001) in the postintervention group. No neonatal deaths were recorded
Lenguerrand et al. (2019) [30]	• Reduction of the proportion of term babies with Apgar < 7 at 5 min	• L4b (patient-level outcome)	• Moderate but non-significant reduction
Sawyer et al. (2019) [29]	• Self-perceived value of training and self-perceived knowledge gains • CPR coordination and appropriate ECPR activation • ECLS survival and survival to discharge	• L1 (reaction) • L3 (behavior/process) • L4b (patient-level outcome)	• Self-perceived value and knowledge gains were improved • Improved adherence to the ECPR activation protocols and faster ECPR activation • ECLS survival and survival to discharge increased, but results were not statistically significant
Balachander et al. (2020) [33]	• IV cannulation success rate and conscious use of aseptic technique • Reduction in BSI, PLABSI, and CLABSI rate	• L3 (behavior/process) • L4b (patient-level outcome)	• Improved IV cannulation success rate and conscious use of aseptic technique • Reduction in BSI, PLABSI, and CLABSI rates
Herrick et al. (2021) [11]	• Increase use of AB and VL • Reduction of overall and severe TIAEs	• L3 (behavior/process) • L4b (patient-level outcome)	• Increased use of AB for at-risk patients (70% vs. 90%) and at intubation (18% vs. 50%); increased VL use (86% vs. 97%) • Reduction of severe TIAEs
Chitkara et al. (2023) [25]	• Survival to discharge without CLD • Other clinical outcomes (intubation, hypothermia, etc.)	• L4b (patient-level outcome) • L4b (patient-level outcome)	• No significant difference in newborns’ survival without CLD between intervention and control groups • No effect of in situ simulation on all secondary outcomes
Studies focused on improvements insystem-level process (Level 4a) and in patient-level outcome (Level 4b)			
Walker et al. (2015) [35]	• Reduction of cesarean delivery • Reduction of hospital-based neonatal mortality	• L4a (system-level process) • L4b (patient-level outcome)	• Decrease of incidence of caesarean sections in intervention hospitals • 40% reduction in neonatal mortality adjusting for baseline differences was apparent at 8 months postintervention
McKeta et al. (2021) [27]	• Reduction of unplanned transfers to the cardiac ICU • Reduction of cardiac arrests and mortality	• L4a (system-level process) • L4b (patient-level outcome)	• Reduction in unplanned transfers to the cardiac ICU • Reduction in cardiac arrests and mortality on the cardiac stepdown unit

*AB* airway bundle, *AHA-PALS* American Heart Academy-Pediatric Advanced Life Support, *AV* audiovisual, *BSI* blood stream infections, *BRE* blinded rater evaluation, *CC* chest compression, *CLABSI* central line associated blood stream infection, *CLD* chronic lung disease, *CPA* cardiopulmonary arrest, *CPR* cardiopulmonary resuscitation, *ECMO* extracorporeal membrane oxygenation, *ECPR* extracorporeal cardiopulmonary resuscitation, *ECLS* extracorporeal life support, *IV* intravenous, *LP* lumbar puncture, *MRSA* methicillin-resistant *Staphylococcus aureus,* *OSCE* Observed Structured Clinical Examination, *PICU* pediatric intensive care unit, *PLABSI* peripheral line–associated blood stream infection, *TIAEs* adverse tracheal intubation-associated events, *VL* video laryngoscope

The included studies were published between 2011 and 2023. Of the 16 studies, nine (56%) were conducted in the USA [9–11, 24–29], two in the UK [30, 31], one in Australia [32], and at least four in low- or middle-income countries (LMICs) [33–36]. Six studies (37%) had a retrospective design [10, 11, 27, 29, 33, 34], while ten (63%) were prospective [9, 24–26, 28, 30–32, 35, 36], and three were RCTs (19%) [9, 30, 35]. The majority of studies (69%, 11/16) were conducted single-center [9–11, 24, 26–29, 31, 33, 36], while five were multicenter [25, 30, 32, 34, 35].

### Study population

Apart from one study involving only nurses [24] and another focusing on pediatric residents [9], all remaining studies were multidisciplinary and included physicians, trainees, fellows, nurses, physiotherapist,s and environmental service workers.

Participant numbers were not consistently reported; among studies that did provide data, sample sizes ranged from 56 to 524 (median 89) healthcare providers.

The program duration ranged from 4 to 60 months (median 32 months). Study contexts were heterogeneous: more than half were carried out in the NICU (56%, 9/16) [11, 25, 26, 30, 32–35], while the others in the PICU. Notably, four of the six studies involving pediatric patients were implemented across both the pediatric emergency department (PED) and PICU settings [10, 27, 28, 31]. Only two studies were performed exclusively in the PICU: one focused on extracorporeal membrane oxygenation (ECMO) support [29], and the other on lumbar puncture skills [9]. The study by Scholtz et al. [24] uniquely addressed a longitudinal intervention (central venous catheter [CVC] dress rehearsal) involving multiple departments.

Intervention types varied: seven studies [10, 27–32] focused on teamwork and early activation of emergency response teams during cardiac events, while five targeted a single technical skill [9, 11, 24, 26, 33]. Detailed characteristics of each study are presented in Table 1.

### Primary and secondary outcomes

The included studies focused on two distinct dimensions of Kirkpatrick Level 4 outcomes: system-level processes (4a) and actual patient health outcomes (4b).

1. Kirkpatrick Level 4a (system-level processes): Five studies specifically addressed organizational efficiency and healthcare delivery processes. Significant improvements were demonstrated by Su et al. [10] through faster ECMO deployment and by Theilen et al. [31] through reductions in PICU bed days and unplanned admissions. Similarly, Walker et al. [35] observed a decrease in caesarean section rates in intervention hospitals, and McKeta et al. [27] reported a reduction in unplanned transfers to the cardiac intensive care unit. In contrast, an RCT by [9] focusing on lumbar puncture skills failed to show a significant reduction in traumatic procedures.
2. Kirkpatrick Level 4b (actual patient outcomes): Direct impacts on patient health were reported in the majority of studies [11, 24–30, 32, 34–36]. In pediatric settings, notable successes included substantial and sustained improvements in cardiopulmonary arrest survival rates [28] and reduced mortality within cardiac stepdown units [27]. In neonatal contexts, SBE led to reduced 24-h neonatal mortality [36], decreased asphyxia-related morbidity [34], and lower rates of severe tracheal intubation-associated events [11]. Furthermore, a reduction in healthcare-associated infections—including MRSA colonization, CLABSI, and PLABSI—was observed [26, 33].
3. Kirkpatrick Levels 1–3 (educational and behavioral foundations): While the primary focus of this review is Level 4, concurrent improvements across lower Kirkpatrick levels served as the foundation for clinical change. Participants across multiple studies [9, 24, 26, 28, 29] reported high self-perceived value, increased confidence, and objective learning gains (Levels 1 and 2), evidenced by better OSCE performance [9] and enhanced psychomotor skills on mannequins [24]. The clinical gains of Level 4 were underpinned by behavioral changes (Level 3) such as consistent use of airway bundles [11], improved hand hygiene [26], and superior coordination and faster deployement during CPR protocols [29].

Despite the shared goal of improving clinical care, the translation from educational success to statistical clinical significance was not universal. In 18% of the studies (3/16), positive trends were observed without reaching statistical significance. For instance, Su et al. [10] noted survival increases that did not reach significance, and an RCT on lumbar puncture skills [9] failed to reduce traumatic clinical procedures despite improved trainee knowledge. Additionally, some studies reported hospital-wide process improvements (4a) without a corresponding reduction in overall consultant review times or specific neonatal clinical outcomes (4b) [25, 31, 32, 34, 35]. These discrepancies highlight that while SBE effectively enhances staff competency and safety culture, its direct, measurable impact on patient mortality and morbidity remains variable.

### Quality assessment

All three RCTs [9, 30, 35] were assessed as having a (RoB) according to the Revised Cochrane RoB 2 tool [21] (Table S3, Supplementary Material 1). For nonrandomized and before–after studies, the ROBINS-I-V2 tool revealed that seven out of 13 studies had a serious RoB, while the remaining six were judged to have a critical RoB (Tables S4 and S5, Supplementary Material 1). To evaluate the consistency of these assessments, interrater reliability was calculated using Cohen’s kappa. The analysis yielded a value of 0.91 (95% CI: 0.86–0.96), indicating almost perfect interrater agreement. All 13 instances of initial disagreement were successfully resolved through consensus with the senior investigator (G.J.).

## Discussion

### Principal finding: the scarcity of high-level evidence

The most significant finding of this systematic review is not the success of specific interventions, but rather the stark scarcity of rigorous Level 4 evidence linking SBE to patient outcomes in pediatric and neonatal critical care. Despite the global adoption of simulation over the past 15 years, our analysis identified only 16 studies meeting our criteria, nearly all of which were limited by a high or critical risk of bias (Tables 1 and 2). Despite the widespread global adaption of SBE and its integration into undergraduate and postgraduate medical curricula, much of the existing SBR remains focused on the educational mechanics of simulation, rather than its measurable impact on clinical care.

Nonetheless, our findings highlight that SBE can positively influence patient outcomes, particularly in well-defined, high-risk clinical areas. While our results indicate that SBE can influence cardiac arrest survival [27, 28] and infection rates [24, 26, 33], these associations are weakened by the lack of randomized designs. Consequently, the uniformly low methodological rigor identified in this review is not a peripheral limitation; it is a fundamental finding that must guide the future of the field.

A key observation is that most of the included studies did not achieve the anticipated clinical improvements, even when some positive trends were observed [27, 35]. This result highlights the impact of SBE on patient outcomes is not guaranteed and is influenced by multiple factors. A major ongoing challenge in this field is the lack of standardized, objective outcome measures, which hinders the consistent evaluation and comparison of the true clinical effectiveness of simulation-based interventions.

### Why outcomes diverge

The rarity of Level 4b (patient-level) evidence is driven by several structural and methodological barriers. First, many simulation interventions are complex, multicomponent “bundles” where isolating the effect of simulation from changes in equipment, staffing, or policy is statistically difficult [16–19, 37, 38]. Second, the “signal-to-noise” ratio in critical care is a major hurdle; because mortality and severe adverse events are (thankfully) relatively rare, studies often lack the massive sample sizes required to achieve statistical power for patient-level outcomes [10]. Finally, there is a translational lag between simulation-acquired competence and institutional change, which standard short-term research designs often fail to capture. A fundamental challenge within the Kirkpatrick model lies in capturing the complex transfer of learning from simulation environments to real-world clinical practice [16]. While SBE can effectively improve individual knowledge and technical skills, translating these improvements into measurable patient outcomes remains difficult and can be influenced by several factors—namely, moderators, mediators, and confounders [16, 17, 19].

While this review focused on quantitative outcomes to map Level 4 impact, we acknowledge that quantitative data alone often fail to capture the “why” behind these divergences. Qualitative simulation research provides vital insights into the socio-cultural and contextual mechanisms—such as psychological safety, hierarchy, and leadership—that determine whether a skill learned in a lab is actually applied at the bedside. Truly answering “how” and “for whom” simulation works requires a synthesis of paradigms. While RCTs establish efficacy, qualitative inquiry explains the nuanced variability and implementation fidelity observed across different clinical contexts.

### Subgroups and contextual variations

This systematic review highlights important key differences in outcomes based on study design and context.

Single-center vs. multicenter studies: Notably, single-center studies were more likely to demonstrate significant improvements in patient outcomes compared to multicenter studies [10, 11, 24, 27–29, 31, 33, 36]. This may be attributed to their clearer focus, precise primary outcomes, and the ability to tailor interventions to specific local needs. These studies often combined microlearning strategies with alignment to specific institutional culture, likely enhancing participant engagement. The SBR model provides a framework for understanding why these outcomes diverge across different contexts. At this stage of the argument, the model is introduced to illustrate that the success of a simulation intervention is not solely dependent on the educational content, but on the alignment of five interconnected elements. The layers of influence that determine clinical impact are as follows: (1) individual competence (technical/nontechnical skills); (2) team dynamics (communication and leadership); (3) institutional culture (psychological safety and administrative support); (4) physical resources (equipment and staffing availability); and (5) system-level integration (adherence to national or local protocols). By viewing research through this lens, it becomes clear why single-center studies—which can more easily align these five layers—often show more significant results than multicenter trials. Furthermore, this model maps directly onto the recommendations in the “ Research implications” section; we argue that future SBR must move beyond measuring Level 1 and 2 outcomes and instead adopt study designs that account for the synergy between these five elements to bridge the gap between training and patient-level (Level 4b) results.

Conversely, several multicenter cases [25, 30, 32, 35] encountered implementation challenges. As illustrated in the THISTLE trial [30], inconsistencies emerged in the qualifications of local trainers and delivery methods, which can obscure the true effectiveness of the program and dilute the intervention’s impact.

High-income (HICs) vs. low- and middle-income countries (LMICs): While most research has been conducted in HICs, evidence from LMICs—particularly when SBE was integrated into national health strategies—demonstrated notable improvements in clinical outcomes, including neonatal mortality [34–36]. This suggests that SBE may be especially impactful in resource-limited settings where it directly addresses critical gaps in essential care.

Clinical settings: NICU, PICU, and PED: Although simulation showed utility across all three settings, its effect on long-term patient survival remains inconsistent. Studies in PICU and PED settings reported significant patient-level benefits such as improved cardiopulmonary arrest survival [31] and reduced unplanned transfers [27, 31]. In contrast, findings from specialized NICU settings were more mixed. One large multicenter NICU study found no significant improvement in survival without chronic lung disease (CLD) following an extensive in situ program [25].

RCTs vs. observational designs: A critical methodological gap persists: the vast majority of studies utilized retrospective or prospective before–after comparisons, with only three employing an RCT or cluster-RCT design [9, 30, 35]. While observational studies—such as those reporting faster ECPR deployment [10] or reduced CLABSI rates [24, 33]—show promising local successes, they remain susceptible to confounding variables and secular trends. In contrast, the more rigorous cluster-RCTs produced more nuanced findings, showing only time-limited reductions in mortality [35] or nonsignificant results [30]. This contrast suggests that current observational evidence may overestimate the impact of simulation, underscoring the critical need for future research to prioritize cluster-RCT designs combined with qualitative process evaluations.

### Implications for practice and policy

To achieve meaningful improvements in system processes and patient outcomes (Levels 4a and 4b), there are several implications for practice and policy. SBE must be implemented under conditions that support its effectiveness. Rather than making broad claims, practitioners and policymakers should focus on enabling success through well-structured, context-sensitive strategies. Effective and feasible SBE programs often share three key features: (1) targeted interventions that address specific, high-impact clinical areas with clearly defined, measurable outcomes [39]; (2) high-intensity training, such as low-dose, high-frequency models, to ensure skill acquisition and retention [40]; and (3) strong integration and support, including leadership engagement and strategies to address and overcome real-world barriers to implementation [41, 42].

### Research implications

The findings of this review highlight several key directions for future research in SBE. To move the field forward, researchers should shift from descriptive studies to theory-driven, mixed-methods designs. We recommend the following:

1. Process evaluations: Integrating qualitative assessments to track how and why interventions succeed or fail during implementation.
2. Mechanism alignment: Ensuring that chosen outcome measures (e.g., time to first shock) are directly aligned with the intended mechanism of change of the simulation (e.g., CPR teamwork), going beyond learner’s knowledge and skill acquisition, and including clinically meaningful patient outcomes.
3. Cluster-randomized trials: Utilizing multicenter, cluster-RCT designs to account for institutional confounding variables while maintaining the “real-world” context of the training.

Finally, adequate sample sizes are essential to detect statistically significant effects and transparent reporting must include not just intervention intensity, but also the qualitative “narrative” of the implementation process to facilitate reproducibility across diverse healthcare settings.

### Limitations

This review has several limitations. First, the search strategy may have introduced retrieval bias, as key databases such as EMBASE, Scopus/Web of Science, PsycINFO, and other indexes focused on LMICs like LILACS and the Global Index Medicus were not included. This likely resulted in an overrepresentation of studies from HICs and may have excluded important, relevant studies from other regions or fields. Additionally, the exclusion of clinical trial registries, abstracts, unpublished trials, and gray literature may have introduced publication bias, potentially overestimating of the effectiveness of simulation-based programs, as studies with positive findings are more likely to be published.

Only 16 studies met the inclusion criteria, encompassing different study designs and outcomes, which precluded a meta-analysis. The heterogeneity in intervention timing, outcome measurement windows, and lack of long-term follow-up further limited the ability to draw definitive conclusions about the impact of SBE on patient outcomes.

Finally, the overall quality of evidence was variable, with several sources at high risk of bias due to issues such as inadequate reporting of randomization procedures, the inability for participants to be blinded, and potential influence from researchers who also acted as facilitators. These limitations underscore the need for a cautious interpretation of the findings.

## Conclusion

This systematic review and critical appraisal highlight that, despite the widespread adoption of SBE in pediatric and neonatal critical care, robust evidence linking it to improved patient outcomes (Kirkpatrick Level 4) remains limited and methodologically inconsistent. Of over 2400 studies screened, only 16 met the inclusion criteria for evaluating patient-level outcomes, revealing a substantial research gap in high-level SBR.

The current literature is dominated by single-center, before–after designs that were most successful when narrowly focused (procedural training, e.g., CLABSI) and team-based emergencies (e.g., cardiac arrest management) but they are susceptible to bias and secular trends. To bridge this translational gap, the next generation of simulation-based research must transition toward rigorous, multicenter, mixed-methods designs that prioritize standardized patient-level metrics. Only through this shift from educational description to critical clinical appraisal can we definitively establish simulation as a primary driver of improved patient outcomes in pediatric and neonatal critical care.

## Supplementary information

Below is the link to the electronic supplementary material.

ESM 1 (DOCX 58.4 KB)

## Data Availability

The datasets used and/or analyzed during the current study are available from the corresponding author upon reasonable request.

## Abbreviations

AB: Airway bundles
BSI: Bloodstream infections
CICU: Cardiac intensive care unit
CLABSI: Central line–associated bloodstream infections
CPA: Cardiopulmonary arrest
CVC: Central venous catheter
ECLS: Extracorporeal life support
ECMO: Extracorporeal membrane oxygenation
ECPR: Extracorporeal cardiopulmonary resuscitation
HICs: High-income countries
IV: Intravenous
LMICs: Low middle-income countries
MRSA: Methicillin-resistant *Staphylococcus aureus*
NICU: Neonatal intensive care unit
No-RCT: Nonrandomized controlled trials
PED: Pediatric emergency department
PICO: Population intervention comparison outcomes
PICU: Pediatric intensive care unit
PLABSI: Peripheral line–associated bloodstream infections
PRISMA: Preferred Reporting Items for Systematic Reviews and Meta-Analyses
RCT: Randomized controlled trials
ROBINS-I-V2: Risk of Bias in Non-randomized Studies of Interventions-Version 2
SBE: Simulation-based education
SBR: Simulation-based research
TIAEs: Tracheal intubation adverse events
VL: Video laryngoscope
