# Supplementary material for: Evaluating the impact of simulation on patient outcomes in pediatric and neonatal critical care: a systematic review and critical appraisal of the evidence
Source: Eur J Pediatr. 2026 Apr 26;185(5):302. doi: 10.1007/s00431-026-06925-3 (PMC13653824; doi:10.1007/s00431-026-06925-3)
Supplement: Supplementary file 1 [file 431_2026_6925_MOESM1_ESM.docx]

**Table S1. Research question according to PICO**

| **Population** | Pediatric/neonatal critical care |
| --- | --- |
| **Intervention** | Simulation-based education (SBE) intervention |
| **Comparison** | traditional/ no training |
| **Outcomes** | patient outcomes |

## **Table S2. Search Strategies**

| Medline | ("Simulation-Based Education" "simulation-based research" OR "Teaching"[Mesh] OR Teaching OR education) AND ("Treatment Outcome"[Mj] OR "Patient Outcome Assessment"[Mesh] OR "Patient Outcome Assessment" OR "Patient Outcome") AND ("Intensive Care Units, Pediatric"[Mesh] OR "Intensive Care Units Neonatal" OR "Intensive Care Units Pediatric" OR NICU OR PICU) |
| --- | --- |
| Cochrane Library | (( OR (teach* OR educat* OR "Simulation-Based Education" OR "simulation-based research"):ti,ab,kw) AND (] OR [mh "Patient Outcome Assessment"] OR ("Patient Outcome Assessment" OR "Patient Outcome" OR "Treatment Outcome"):ti,ab,kw) AND ([mh "Intensive Care Units, Pediatric"] OR [mh "Intensive Care Units, Neonatal"] OR (NICU OR PICU OR "Intensive Care Units Pediatric" OR "Intensive Care Units Neonatal"):ti,ab,kw)) |
| CINAHL | ((MH "Teaching+") OR TI (teach* OR educat* OR "Simulation-Based Education" OR "simulation-based research") OR AB (teach* OR educat* OR "Simulation-Based Education" OR "simulation-based research")) AND ((MM "Treatment Outcomes+") OR MH "Outcomes (Health Care)+" OR TI ("Patient Outcome Assessment" OR "Patient Outcome" OR "Treatment Outcome") OR AB ("Patient Outcome Assessment" OR "Patient Outcome" OR "Treatment Outcome")) AND ((MH "Intensive Care Units, Pediatric+") OR MH "Intensive Care Units, Neonatal+" OR TI (NICU OR PICU OR "Intensive Care Units Pediatric" OR "Intensive Care Units Neonatal") OR AB (NICU OR PICU OR "Intensive Care Units Pediatric" OR "Intensive Care Units Neonatal")) |
| ERIC | ((DE "Simulation" OR DE "Teaching Methods") OR TI (teach* OR educat* OR "Simulation-Based Education" OR "simulation-based research") OR AB (teach* OR educat* OR "Simulation-Based Education" OR "simulation-based research")) AND ((MM "Outcomes of Treatment" OR DE "Outcome Measures") OR TI ("Patient Outcome Assessment" OR "Patient Outcome" OR "Treatment Outcome") OR AB ("Patient Outcome Assessment" OR "Patient Outcome" OR "Treatment Outcome")) AND (TI (NICU OR PICU OR "Intensive Care Unit*" OR "Neonatal Intensive Care" OR "Pediatric Intensive Care") OR AB (NICU OR PICU OR "Intensive Care Unit*" OR "Neonatal Intensive Care" OR "Pediatric Intensive Care")) |

**Table S3. Quality assessment for randomized controlled trials on the primary outcome (RoB 2)**

**Questions’ domains:**

Domain 1: Risk of bias arising from the randomization process

Domain 2: Risk of bias due to deviations from the intended interventions (effect of assignment to intervention)

Domain 3: Risk of bias due to missing outcome data

Domain 4: Risk of bias in measurement of the outcome

Domain 5: Risk of bias in selection of the reported result

| **Study** | | **Rob arising from**  **the randomization process** | | | **RoB J.** | **Rob due to the deviations**  **from the intended intervention**  **(effect of assignment to intervention)** | | | | | | | **RoB J.** |
| --- | --- | --- | --- | --- | --- | --- | --- | --- | --- | --- | --- | --- | --- |
| **Author** | **Year** | **Q1.1** | **Q1.2** | **Q1.3** |  | **Q2.1** | **Q2.2** | **Q2.3** | **Q2.4** | **Q2.5** | **Q2.6** | **Q2.7** |  |
| Kessler | 2011 | Y | NI | N | Low risk | PY | PY | Y | PY | Y | Y | NA | Some concerns |
| Walker | 2015 | Y | NI | PN | Low risk | Y | Y | Y | PY | NI | Y | NA | High risk |
| Lenguerrand | 2019 | Y | Y | N | Low risk | Y | Y | Y | Y | N | Y | NA | High risk |

| **Study** | | **Rob due to**  **missing outcome data** | | | | **RoB J.** | **Rob in measurement**  **of the outcome** | | | | | **RoB J.** | **Rob in the selection of the reported result** | | | **RoB J.** | **Overall RoB J.** |
| --- | --- | --- | --- | --- | --- | --- | --- | --- | --- | --- | --- | --- | --- | --- | --- | --- | --- |
| **Author** | **Year** | **Q3.1** | **Q3.2** | **Q3.3** | **Q3.4** |  | **Q4.1** | **Q4.2** | **Q4.3** | **Q4.4** | **Q4.5** |  | **Q5.1** | **Q5.2** | **Q5.3** |  |  |
| Kessler | 2011 | N | Y | NA | PY | Some concerns | Y | Y | NA | PY | PY | High risk | Y | N | PN | Low risk | High risk |
| Walker | 2015 | Y | NA | NA | NA | Some concerns | N | N | PY | PY | NI | High risk | NI | N | N | Low risk | High risk |
| Lenguerrand | 2019 | Y | Y | NA | PN | Low risk | N | N | Y | NA | NA | High risk | Y | N | N | Low risk | High risk |

**Abbreviations:** RoB J.: Risk-of-bias judgement; Y: yes; N: no; NA: not applicable; NI: no information; PN: probably no; PY: probably yes

### **Table S4. Quality assessment for non-randomized controlled trials on the primary outcome variant A- domain 1 (ROBINS-I-V2)**

**Questions’ domains:**

Domain 1: Risk of bias due to confounding – variant A (only baseline confounding needs to be addressed)

Domain 2: Risk of bias in classification of intervention

Domain 3: Risk of bias in selection of participants into the study (or into the analysis)

Domain 4: Risk of bias due to deviations from intended interventions

Domain 5: Risk of bias due to missing data

Domain 6: Risk of bias in measurement of the outcome

Domain 7: Risk of bias in selection of the reported result

| **Study** | | **Rob due to confounding – variant A** | | | | **ROBINS-I-V2** | **Rob in classification of intervention** | | | | | **ROBINS-I-V2** |
| --- | --- | --- | --- | --- | --- | --- | --- | --- | --- | --- | --- | --- |
| **Author** | **Year** | **Q1.1** | **Q1.2** | **Q1.3** | **Q1.4** |  | **Q2.1** | **Q2.2** | **Q2.3** | **Q2.4** | **Q2.5** |  |
| Andreatta | 2011 | PY | Y | N | N | Low Rob | NA | NA | PY | N | NA | Serious Rob |
| Scholtz | 2013 | WN | Y | N | Y | Serious Rob | N | NA | Y | N | Y | Low Rob |
| Su | 2014 | WN | Y | N | Y | Serious Rob | N | NA | Y | N | Y | Low Rob |
| Mduma | 2015 | SN | NA | NA | Y | Critical Rob | N | NA | Y | N | Y | Low Rob |
| Gibbs | 2017 | SN | NA | NA | Y | Critical Rob | N | NA | Y | N | Y | Low Rob |
| Theilen | 2017 | SN | NA | NA | Y | Critical Rob | N | NA | Y | N | Y | Low Rob |
| Schmidt | 2018 | SN | NA | NA | Y | Critical Rob | N | NA | Y | N | Y | Low Rob |
| Kumar | 2018 | Y | PY | NI | PN | Low Rob | N | NA | Y | N | Y | Low Rob |
| Sawyer | 2019 | Y | PY | NI | PN | Low Rob | N | NA | Y | N | Y | Low Rob |
| Balachander | 2020 | NI | NI | N | Y | Critical Rob | N | NA | Y | N | Y | Low Rob |
| Herrick | 2021 | PY | Y | N | N | Low Rob | N | NA | Y | N | Y | Low Rob |
| McKeta | 2021 | SN | Y | N | Y | Critical Rob | N | NA | Y | N | Y | Low Rob |

| **Study** | | **Rob in selection of participants into the study** | | | | | | | | | | **ROBINS-I-V2** |
| --- | --- | --- | --- | --- | --- | --- | --- | --- | --- | --- | --- | --- |
| **Author** | **Year** | **Q3.1** | **Q3.2** | **Q3.3** | **Q3.4** | **Q3.5** | **Q3.6** | **Q3.7** | **Q3.8** | **Q3.9** | **Q3.10** |  |
| Andreatta | 2011 | NA | NA | PY | NA | N | NA | NA | NA | NA | NA | Moderate Rob |
| Scholtz | 2013 | N | NA | N | N | N | NA | NA | N | N | N | Serious Rob |
| Su | 2014 | N | NA | N | NA | N | NA | NA | NA | NA | NA | Moderate Rob |
| Mduma | 2015 | N | NA | N | PN | N | NA | NA | N | N | PN | Serious Rob |
| Gibbs | 2017 | N | NA | N | PN | N | NA | NA | N | N | PN | Serious Rob |
| Theilen | 2017 | N | NA | N | PN | N | NA | NA | N | N | PN | Serious Rob |
| Schmidt | 2018 | N | NA | N | PN | N | NA | NA | N | N | PN | Serious Rob |
| Kumar | 2018 | N | NA | N | PN | N | NA | NA | PY | NA | NA | Moderate Rob |
| Sawyer | 2019 | N | NA | N | PN | N | NA | NA | Y | NA | NA | Moderate Rob |
| Balachander | 2020 | N | NA | N | N | Y | N | NA | NA | NA | NA | Serious Rob |
| Herrick | 2021 | N | NA | N | Y | N | NA | NA | Y | NA | NA | Moderate Rob |
| McKeta | 2021 | N | NA | Y | NA | N | NA | NA | NA | NA | NA | Low risk |

| **Study** | | **Rob due to deviations from intended interventions** | | | | | **ROBINS-I-V2** | **Rob due to missing data** | | | | | | | | | | | **ROBINS-I-V2** |
| --- | --- | --- | --- | --- | --- | --- | --- | --- | --- | --- | --- | --- | --- | --- | --- | --- | --- | --- | --- |
| **Author** | **Year** | **Q4.1** | **Q4.2** | **Q4.3** | **Q4.4** | **Q4.5** |  | **Q5.1** | **Q5.2** | **Q5.3** | **Q5.4** | **Q5.5** | **Q5.6** | **Q5.7** | **Q5.8** | **Q5.9** | **Q5.10** | **Q5.11** |  |
| Andreatta | 2011 | PY | N | N | NA | Y | Low Rob | NA | Y | Y | NA | NA | NA | NA | NA | NA | NA | NA | Low Rob |
| Scholtz | 2013 | Y | N | N | NA | Y | Low Rob | Y | Y | Y | NA | NA | NA | NA | NA | NA | NA | NA | Low Rob |
| Su | 2014 | Y | N | N | NA | Y | Low Rob | Y | Y | NA | NA | NA | NA | NA | NA | NA | NA | NA | Low Rob |
| Mduma | 2015 | Y | N | N | NA | SN | Serious Rob | Y | Y | Y | NA | NA | NA | NA | NA | NA | NA | NA | Low Rob |
| Gibbs | 2017 | Y | N | PN | NA | SN | Serious Rob | Y | Y | Y | NA | NA | NA | NA | NA | NA | NA | NA | Low Rob |
| Theilen | 2017 | Y | N | N | NA | SN | Serious Rob | Y | Y | Y | NA | NA | NA | NA | NA | NA | NA | NA | Low Rob |
| Schmidt | 2018 | Y | NI | NI | PY | SN | Serious Rob | Y | Y | Y | NA | NA | NA | NA | NA | NA | NA | NA | Low Rob |
| Kumar | 2018 | Y | PN | NI | Y | Y | Serious Rob | Y | Y | Y | NA | NA | NA | NA | NA | NA | NA | NA | Low Rob |
| Sawyer | 2019 | Y | PN | NI | Y | Y | Serious Rob | Y | Y | Y | NA | NA | NA | NA | NA | NA | NA | NA | Low Rob |
| Balachander | 2020 | Y | N | N | NA | Y | Low Rob | Y | Y | Y | NA | NA | NA | NA | NA | NA | NA | NA | Low Rob |
| Herrick | 2021 | PN | NA | NA | NA | Y | Low Rob | Y | Y | Y | NA | NA | NA | NA | NA | NA | NA | NA | Low Rob |
| McKeta | 2021 | PN | NA | NA | NA | SN | Serious Rob | Y | Y | Y | NA | NA | NA | NA | NA | NA | NA | NA | Low Rob |

| **Study** | | **Rob in measurement of the outcome** | | | **ROBINS-I-V2** | **Rob in selection**  **of the reported result** | | | | **ROBINS-I-V2** | **Overall Rob** |
| --- | --- | --- | --- | --- | --- | --- | --- | --- | --- | --- | --- |
| **Author** | **Year** | **Q6.1** | **Q6.2** | **Q6.3** |  | **Q7.1** | **Q7.2** | **Q7.3** | **Q7.4** |  |  |
| Andreatta | 2011 | N | Y | N | Low Rob | NI | N | N | N | Low Rob | Serious Rob |
| Scholtz | 2013 | N | Y | N | Low Rob | NI | N | N | N | Low Rob | Serious Rob |
| Su | 2014 | N | Y | N | Low Rob | NI | N | N | N | Low Rob | Serious Rob |
| Mduma | 2015 | PY | Y | SY | Serious Rob | NI | N | N | N | Low Rob | Critical Rob |
| Gibbs | 2017 | Y | Y | SY | Serious Rob | NI | N | N | N | Low Rob | Critical Rob |
| Theilen | 2017 | PY | Y | PN | Serious Rob | NI | N | N | N | Low Rob | Critical Rob |
| Schmidt | 2018 | PY | Y | SY | Serious Rob | NI | N | N | N | Low Rob | Critical Rob |
| Kumar | 2018 | Y | Y | SY | Serious Rob | Y | N | N | N | Low Rob | Serious Rob |
| Sawyer | 2019 | PN | Y | N | Low Rob | NI | N | N | N | Low Rob | Serious Rob |
| Balachander | 2020 | Y | Y | SY | Serious Rob | NI | N | N | N | Low Rob | Critical Rob |
| Herrick | 2021 | Y | Y | SY | Serious Rob | PN | N | N | N | Low Rob | Serious Rob |
| McKeta | 2021 | N | Y | N | Low Rob | NI | N | Y | N | Serious Rob | Critical Rob |

**Abbreviations:** ROBINS-I-V2: Risk Of Bias In Non-randomised Studies - of Interventions- Version 2; Rob: Risk of Bias; Y: yes; N: no; NA: not applicable; NI: no information; PN: probably no; PY: probably yes; SN: no, and uncontrolled confounding was probably substantial; WN: no, but uncontrolled confounding was probably not substantial

### **Table S5. Quality assessment for non-randomized controlled trials on the primary outcome variant B – domain 1 (ROBINS-I-V2)**

**Questions’ domains:**

Domain 1: Risk of bias due to confounding – variant B (the analysis was based on splitting participants’ follow up time according to intervention received, so both baseline and time-varying) confounding need to be addressed)

Domain 2: Risk of bias in classification of intervention

Domain 3: Risk of bias in selection of participants into the study

Domain 4: Risk of bias due to deviations from intended interventions

Domain 5: Risk of bias due to missing data

Domain 6: Risk of bias in measurement of the outcome

Domain 7: Risk of bias in selection of the reported result

| **Study** | | **Rob due to confounding – variant B** | | | | | **ROBINS-I-V2** | **Rob in classification of intervention** | | | | | **ROBINS-I-V2** |
| --- | --- | --- | --- | --- | --- | --- | --- | --- | --- | --- | --- | --- | --- |
| **Author** | **Year** | **Q1.1** | **Q1.2** | **Q1.3** | **Q1.4** | **Q1.5** |  | **Q2.1** | **Q2.2** | **Q2.3** | **Q2.4** | **Q2.5** |  |
| Chitkara | 2023 | Y | WN | Y | NA | PY | Serious Rob | N | NA | Y | N | Y | Low Rob |

| **Study** | | **Rob in selection of participants into the study** | | | | | | | | | | **ROBINS-I-V2** |
| --- | --- | --- | --- | --- | --- | --- | --- | --- | --- | --- | --- | --- |
| **Author** | **Year** | **Q3.1** | **Q3.2** | **Q3.3** | **Q3.4** | **Q3.5** | **Q3.6** | **Q3.7** | **Q3.8** | **Q3.9** | **Q3.10** |  |
| Chitkara | 2023 | N | NA | N | Y | Y | Y | Y | N | N | PY | Serious Rob |

| **Study** | | **Rob due to deviations from intended interventions** | | | | | **ROBINS-I-V2** | **Rob due to missing data** | | | | | | | | | | | **ROBINS-I-V2** |
| --- | --- | --- | --- | --- | --- | --- | --- | --- | --- | --- | --- | --- | --- | --- | --- | --- | --- | --- | --- |
| **Author** | **Year** | **Q4.1** | **Q4.2** | **Q4.3** | **Q4.4** | **Q4.5** |  | **Q5.1** | **Q5.2** | **Q5.3** | **Q5.4** | **Q5.5** | **Q5.6** | **Q5.7** | **Q5.8** | **Q5.9** | **Q5.10** | **Q5.11** |  |
| Chitkara | 2023 | PN | NA | NA | NA | Y | Low Rob | Y | PN | PN | Y | Y | WN | NA | NA | NA | NA | N | Serious Rob |

| **Study** | | **Rob in measurement of the outcome** | | | **ROBINS-I-V2** | **Rob in selection**  **of the reported result** | | | | **ROBINS-I-V2** | **Overall Rob** |
| --- | --- | --- | --- | --- | --- | --- | --- | --- | --- | --- | --- |
| **Author** | **Year** | **Q6.1** | **Q6.2** | **Q6.3** |  | **Q7.1** | **Q7.2** | **Q7.3** | **Q7.4** |  |  |
| Chitkara | 2023 | N | Y | WN | Moderate Rob | NI | N | N | N | Low Rob | Serious Rob |

**Abbreviations:** ROBINS-I-V2: Risk Of Bias In Non-randomised Studies - of Interventions- Version 2; Rob: Risk of Bias; Y: yes; N: no; NA: not applicable; NI: no information; PN: probably no; PY: probably yes; WN: no, but uncontrolled confounding was probably not substantial
